# Morphological variations of intra-testicular arterial vasculature in bovine testis - a corrosion casting study

**DOI:** 10.1186/s12917-015-0580-9

**Published:** 2015-10-15

**Authors:** Michał Polguj, Grzegorz Wysiadecki, Michał Podgórski, Jacek Szymański, Katarzyna Olbrych, Łukasz Olewnik, Mirosław Topol

**Affiliations:** Department of Angiology, Interfaculty Chair of Anatomy and Histology, Medical University of Łódź, Narutowicza 60, Łódź, 90-136 Poland; Department of Normal Anatomy, Interfaculty Chair of Anatomy and Histology, Medical University of Łódź, Narutowicza 60, Łódź, 90-136 Poland; Department of Normal and Clinical Anatomy, Interfaculty Chair of Anatomy and Histology, Medical University of Łódź, Narutowicza 60, Łódź, 90-136 Poland; Veterinary Division, Department of Morphological Science, Warsaw University of Life Sciences, Nowoursynowska 159, 02-776 Warsaw, Poland

**Keywords:** Bovine testis, Angioarchitecture, Intra-testicular arteries, Classification

## Abstract

**Background:**

Proper blood supply is necessary for the physiological function of every internal organ. The article offers the first classification of the bovine intra-testicular arteries. A corrosive study focused on the intra-testicular arterial vasculature was performed on 40 bovine testes. The vessels were analyzed accurately using MultiScanBase v.18.02 software.

**Methods:**

A corrosive study focused on the intra-testicular arteries was performed on 40 bovine testes. The vessels were analyzed accurately using MultiScanBase v.18.02 software.

**Result:**

In bulls, the centripetal arteries tended to run straight to the mediastinal region, where they form knot-like vascular structures. Those structures are the origin for centrifugal recurrent branches, running peripherally. However, three basic types of intra-testicular arterial vasculature were noted. Type I had centrifugal, recurrent branches, running peripherally towards the surface of the testis but did not reach the tunica albuginea. Type II exhibited centrifugal, recurrent branches running more horizontally than type I. Type III is the most heterogeneous type, composed of other variform types of arteries not classified as type I or type II. Type II was most commonly observed as a vascular conglomerate of intra-testicular arteries within the arterial network of the mediastinum testis. In type III, artery diameter was significantly smaller than observed in types I and II (*p* < 0.01). Types I and II did not differ between each other regarding artery diameter (*p* > 0.05).

**Conclusion:**

Variations of the intra-testicular arterial vasculature in bovine testis may suggest that particular types of vessels play different physiological roles. The most common type of intra-testicular artery comprising the arterial network of the mediastinum testis was type II.

## Background

Proper blood supply is necessary for the physiological function of every internal organ. As the testes are particularly sensitive to the influence of external factors, even transient ones, minor episodes of ischaemia may lead to functional disturbances [1–3]. Therefore, knowledge of the vascular network of the organ is of great clinical importance.

Contrary to numerous comprehensive descriptions of extra-testicular vessels, a search of extant literature reveals no complete description of the inner vascular complex based on adequate quantitative and qualitative material [4–7]. In particular, no detailed descriptions exist of the morphological variations of the intra-testicular arterial vasculature. Therefore, the challenge of the study was to gather, analyze and photographically record a large number of specimens, thus making a potentially valuable contribution to the understanding of the morphology and function of testicular blood vessels. Modern endocast techniques using highly penetrative synthetic fillings allow detailed descriptions to be made of the arterial network of the testes [5, 8–10]. The present study is the first to use image analysis software to create a precise description of the diameter and length of the vessels; earlier observations of intra-testicular anatomy have been only macroscopic and not supported by any calculations. This article presents the first classification of bovine intra-testicular arterial vasculature, which may be used in future investigations of the testis. To our knowledge, such a study has never been published before.

## Methods

### General information

Forty bovine testes (*Bos primigenius, f. taurus* - Polish black and white Holstein–Friesian breed) were included in the study. All investigations were performed in the Department of Angiology and the Department of Normal and Clinical Anatomy, Medical University of Łódź. The research project and its procedures were approved by the Medical University of Łódź Bioethics Commission (Protocol No. RNN/120/07/KE). The specimens comprised the gonad and its coverings, collected immediately after slaughter by a butcher in MarcUrb Abattoir, Szydlowiec, Poland from 2006 to 2010. The total number of males from which the testes were collected was 38. The bulls were aged from 2.5 to 3.5 years, and hence, were after pubescence. Due to the technical protocol of MarcUrb Abattoir, no attempt was made to match morphological studies to the age of the donor or position of the gonad (right or left). The criteria of exclusion included any macroscopically observed sign of pathology.

### Detailed description of the method

The testicular artery in the proximal part of the spermatic cord was isolated and prepared so as to allow access to the vessel. A corrosive cast of the vessels was obtained by the following process: NaCl solution (0.9 %) was injected into the testicular artery and perfusion was maintained for 10–20 s in order to flush out possible clots. Saline perfusion was followed by injection of 10 ml of 3 % glutaraldehyde solution, in cacodylate buffer (pH 7.4). The testicular artery was then filled with Plastogen G (Plasto-Schmidt, Speyer, Germany), and the resin was stained with red pigment. Next, the gonad was left for 24 h in warm (20 °C) water to toughen the resin.

After toughening, the specimen was placed into 40 % KOH solution (50 °C) for 24 h to dissolve the organic parts. The remnants of the dissolved tissues were removed from the specimen by continuous flushing with warm water for 24 h. The cleaning process was completed by a quick wash with water and a small amount of standard washing liquid, followed by a final flush of distilled water. The cast was later dried out by air flow at room temperature for an appropriate time.

The corrosive casts of intra-testicular arteries were examined visually by a SteREO Discovery.V8 stereoscopic binocular microscope (Carl Zeiss MicroImaging GmbH, Gettingen, Germany, magnification 10–80x). The digital photographic images were saved in TIFF (Tagged Image File Format) format, and were later digitally transformed using CorelDRAW Graphics Suite 12 software (Corel Corporation, Ottawa, Canada).

### Method of measurements

For normalization of measurements, photographic documentation was obtained as follows. The camera and cast specimen were placed in a standardized position using a scale, with the cast specimen fixed with an adjustable clamp and ring stand at a set distance from the camera. The measurements of the intra-testicular arteries were taken from digital photographic documentation processed through MultiScanBase 18.03 software (Computer Scanning System II, Warsaw, Poland). The usefulness and correctness of this method were confirmed in a previous study [7, 10].

The following measurements of the intra-testicular arteries were defined and performed:

1. LCpA (Length of the Centripetal Arteries) - measurements of the centripetal artery taken in a longitudinal plane from beginning (from the extra-testicular artery of the tunica albuginea) to end (the start of the knot-like vascular structures in the vicinity of the mediastinum area) (Fig. 1).

**Fig. 1:**
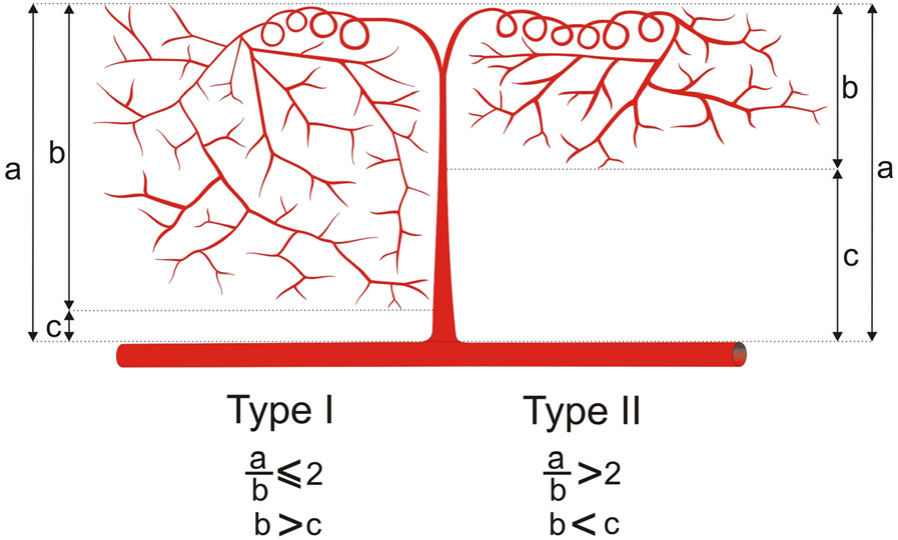
Schematic arrangements of measurements of the intra-testicular arteries: ***a*** - length of the centripetal arteries (LCpA), ***b*** - length of the centrifugal arteries (LCfA)
2. LCfA (Length of the Centrifugal Arteries) - measurements of the centrifugal artery taken in a longitudinal plane from beginning (the end of the knot-like vascular structures in the vicinity of the mediastinum area) to end (Fig. 1).
3. MD - (Maximal Diameter) - the maximal internal diameter of the artery measured between opposite edges of the cast perpendicular to its long axis.

### Statistical analysis

Data analysis was performed using Statistica 10 software (StatSoft Polska, Krakow, Poland). The Shapiro-Wilk test was used to confirm whether the distribution of continuous variables was normal. Difference in diameter of vessel of each type was compared between types by means of the Kruskal-Wallis analysis of variance with dedicated *post hoc* tests. The mean, standard deviation (SD) and minimum and maximum for continuous variables are used as descriptive statistics.

## Results

### General observation

**Fig. 2:**
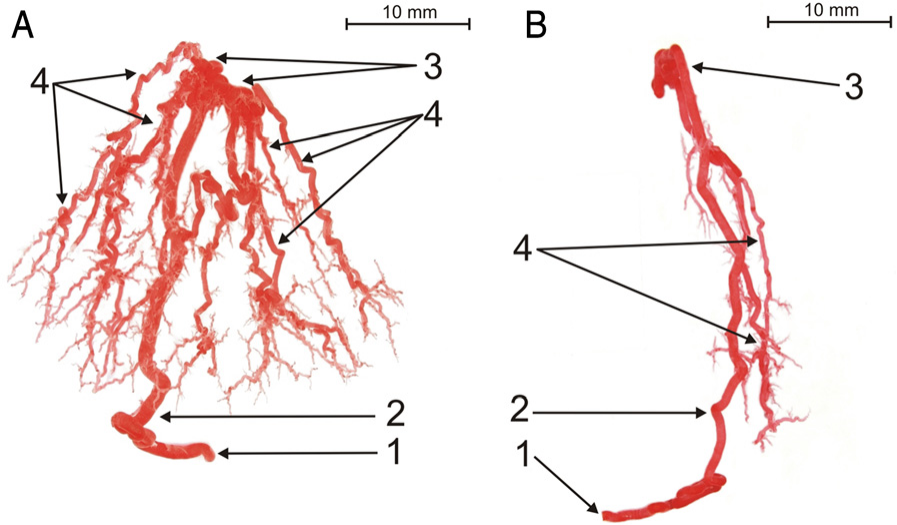
Corrosive cast of bovine testicular arteries (type I of intra-testicular artery: **a** - branched subtype; **b** - tight subtype): 1 - extra-testicular artery, 2 - centripetal arteries (CpA), 3 - knot-like vascular structures (K-L), 4 - centrifugal arteries (CfA)

**Fig. 3:**
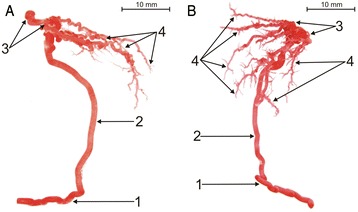
Corrosive cast of bovine testicular arteries (type II of intra-testicular artery: **a** - elongated subtype; **b** - branched subtype): 1 - extra-testicular artery, 2 - centripetal arteries (CpA), 3 - knot-like vascular structures (K-L), 4 - centrifugal arteries (CfA)

**Fig. 4:**
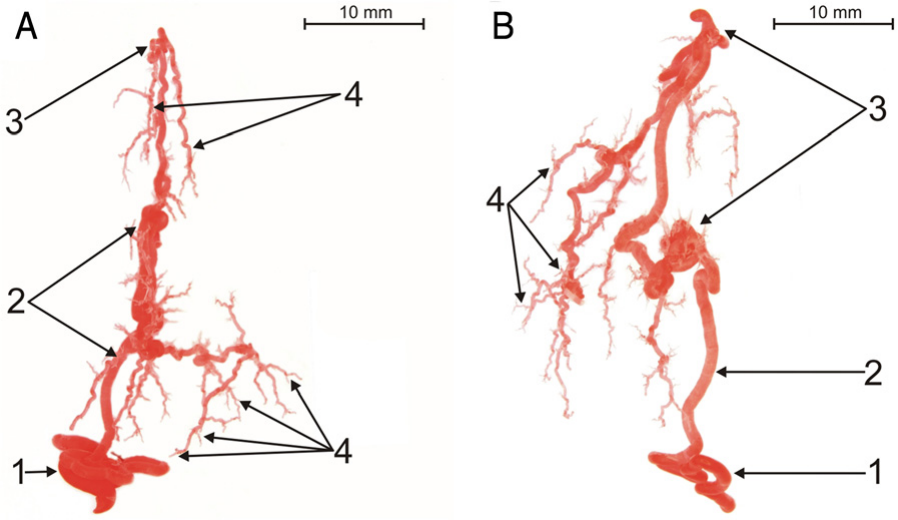
Corrosive cast of bovine testicular arteries (type III of intra-testicular artery; **a** - first subtype; **b** - second subtype): 1 - extra-testicular artery, 2 - centripetal arteries (CpA), 3 - knot-like vascular structures (K-L), 4 - centrifugal arteries (CfA)

Three main types of intra-testicular arterial vasculature were identified in the bovine testis: centripetal, knot-like and centrifugal. Centripetal arteries ran straight from the arteries of the tunica albuginea to the mediastinal region, where they formed knot-like vascular structures. Those structures were the origin for centrifugal, recurrent branches, running peripherally, which did not reach the surface of the testis. The branching of the centrifugal arteries resulted in a tree-like network of one to four generations. In rare cases, knot-like vascular structures lay on the course of a centripetal artery, but never on the course of a centrifugal artery. Centripetal arteries did not anastomose with their branches and formed independent “tree-like arrangements” (Figs. 2, 3, and 4).

### Morphological variations of intra-testicular arterial vasculature

A thorough macroscopic examination of the corrosive casts allowed three types of intra-testicular arterial vasculature to be distinguished, which were present in every specimen. The types differ with respect to their topography and relationship with the intra-testicular arterial vessels.

### Type I of intra-testicular arterial vasculature

The first type (65.1 %) of intra-testicular arteries run straight from extra-testicular arteries (rami tunicales minores and majores) towards the mediastinal region, where they form 2 to 5 knot-like vascular structures. Those structures are also the origin for centrifugal, recurrent branches, running peripherally, which do not reach the surface of the testis. The centrifugal arteries are divided, forming a tree-like network from one to four generations. Its form might be branched (Fig. 2a) or tight (Fig. 2b). The quantitative analysis indicated that LCfA was greater than half of the LCpA (LCpA/LCfA < 2) (Figs. 1, 2).

### Type II of intra-testicular arterial vasculature

In the second type of intra-testicular arterial vasculature (19.4 %), the centripetal arteries run straight from the rami tunicales minores and majores towards the central part of the organ. At the mediastinal region, they form between 4 and 8 knot-like vascular structures. Those structures acted as the origins for centrifugal, recurrent branches, running more longitudinally than the centrifugal branches of type I. Its form may be elongated (Fig. 3a) or branched (Fig. 3b). The quantitative analysis found the LCpA to be at least twice as wide as the LCfA in this type (LCpA/LCfA ≥ 2) (Figs. 1, 3).

### Type III of intra-testicular arterial vasculature

The third type of intra-testicular arterial vasculature (35 %) was the most heterogeneous. It includes such other variform types of arteries as centrifugal arteries arising directly from centripetal arteries (Fig. 4a), or knot-like vascular structures lying on the course of centripetal arteries (Fig. 4b).

### Statistical analysis of diameter of intra-testicular arteries

**Fig. 5:**
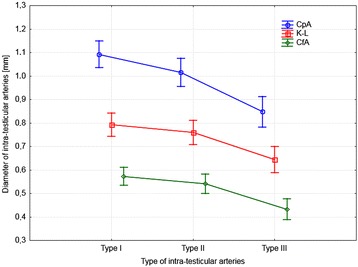
Differences in mean diameter of intra-testicular arteries between three types of vasculature. CpA - centripetal arteries; K-L - knot-like vascular structures; CfA - centrifugal arteries

The Kruskal-Wallis one-way analysis of variance found that the diameter of the intra-testicular arteries was significantly smaller in type III than types I and II (*p* < 0.01). Types I and II did not differ between each other as far as arterial diameter was concerned (*p* > 0.05) (Fig. 5). Detailed measurements of the diameter of intra-testicular arteries are presented in Table 1.

**Table 1:** Diameters of the casts of the intra-testicular bovine arteries

Type of intra-testicular artery	Name of artery	Mean	SD	Min.-Max.
Type I	Centripetal artery	1.09	0.23	0.7–1.57
	‘Knot-like’	0.79	0.18	0.39–1.22
	Centrifugal artery	0.57	0.14	0.34–0.9
Type II	Centripetal artery	1.02	0.14	0.73–1.23
	‘Knot-like’	0.76	0.15	0.51–1.14
	Centrifugal artery	0.54	0.11	0.37–0.8
Type III	Centripetal artery	0.85	0.15	0.49–1.02
	‘Knot-like’	0.64	0.12	0.39–0.94
	Centrifugal artery	0.43	0.11	0.29–0.65

Min.-Max. minimum-maximum, SD standard deviation. Number of gonads – 40. Vessels Mean (mm) SD (mm) Min–Max (mm)

## Discussion

Although Hees et al. [8, 11] and Hundeiker [12, 13] have already described the presence of centripetal, centrifugal and knot-like vessels, they do not discuss the types of intra-testicular arterial vasculature. In contrast, the present study describes the morphological variations of the arteries inside the testis in a detailed way. According to this classification, the arterial network of the mediastinum testis is formed mostly by type II intra-testicular arteries, which run more longitudinally than type I intra-testicular arteries and have almost twice as many knot-like structures. The centrifugal branches of the type II intra-testicular arterial vasculature are short but are divided like type I into between one and four generations. Our findings indicate that type III intra-testicular arteries, with centrifugal branches arising half way along the course of the centripetal arteries, are typically complementary to type II.

Similar to bulls, the centripetal arteries of dogs, stallions, buffalo and rams run straight from the arterial network of the tunica albuginea to the mediastinal region, where they form centrifugal, recurrent branches, running peripherally, which do not reach the surface of the testis [2, 14–16]. However, in the common tree shrew (*Tupalia glis*), the testicular artery emerges from the pampiniform plexus and approaches the gonad at the rostro-medial pole. It then courses caudally along the medial border, then rostrally along the lateral border, and penetrates into the parenchyma near the rostral pole. While curving on the lateral border of the testis, it gives off 4–5 parenchymal branches penetrating perpendicularly into the testicular tissue [9].

Significant differences exist with regard to human and bovine intra-testicular arterial topography [17, 18]. Presumably this is based on differences in mediastinum testis topography present in the two species: central in bovine, peripheral in human. In humans, the intra-testicular arteries originate and run in straight lines centripetally from the tunica albuginea arterial network, and centrifugally from the mediastinum testis network. The centripetal arteries come off the tunica albuginea and run straight towards the mediastinum testis. The centrifugal arteries, running parallel to the centripetal ones, lead off from the mediastinum testis and head towards the tunica albuginea. As a consequence, the blood supply of the testicular tissue is provided by two relatively independent sources.

In bulls, the intra-testicular arteries come off the tunica albuginea arterial network (rami tunicales majores and minores) perpendicularly and pass towards the mediastinum testis, running centripetally. Some of them reach the mediastinum testis and form screw-like helicoids or knot-like vascular conglomerations. Each conglomeration is an originating structure for between 3 and 20 centrifugal arteries parallel to the centripetal ones. The centrifugal arteries do not anastomose with the arterial network of the tunica albuginea. As a consequence, the blood supply of the testicular tissue is provided by one consequent system of arterial vessels [3, 8, 11–13].

In the bull gonad, the highly twisted arrangement of alternating segments straight from the knot-like shape arteries may facilitate the passage of blood and lymph from the centrally located mediastinum to the peripheral part of the organ. No such arrangements were observed in the gonads of a man with an eccentrically located mediastinum [17, 18].

Godinho et al. [19] suggest that the convolutions in the capsular and intra-testicular arteries in ruminants may increase the effective surface area of the arteries to allow exchange of heat and metabolites with the extensive network of juxtaposed centrifugal and capsular veins. Dhinjgra [20] proposes that the coilings of the arteries may also act as a pulse eliminator and a damping mechanism for arterial blood flow. Additionally, Hess et al. [11] propose that the functional significance of the knot-like vascular structures in mediastinum is associated with their location, i.e., where the pulse wave passes the mediastinum. These pulse waves, together with the contractility-flexible fibers of the mediastinum, may support the drainage of lymph towards the surface of the testis. Moreover, arterial conglomerates may facilitate the passage of blood from the intra-testicular veins into the extra-testicular veins [8, 11].

There are several hypotheses concerning the physiological role of the winding and straight segments of the intra-testicular arteries, as well as the arterial conglomerations inside the mediastinum of the bovine testis [9, 17, 18, 20]. However, the absence of these structures in the human testis prompts speculation. It is possible that the winding course of the arteries in bulls compensates for the shorter length of the terminal branches of the testicular artery compared with those of humans. If this is the case, its main role would be to decrease arterial pressure inside the gonad. It is possible that conglomeration of arteries, as in bulls, and a vascular network, as in humans, are two alternative ways for efficient thermoregulation and metabolite exchange with the venous vessels.

## Conclusion

Variations of intra-testicular arteries in the bovine testis may suggest that particular types of vessels play different physiological roles. The most common type of intra-testicular arterial vasculature comprising the arterial network of the mediastinum testis was type II. The diameter of type III intra-testicular arteries was found to be significantly smaller than that of types I and II.

## Abbreviations

KOH: Potassium hydroxide
LCpA: Length of the centripetal arteries
LCfA: Length of the centrifugal arteries
MD: Maximal diameter
Min.-Max.: Minimum-maximum
NaCl: Sodium chloride
SD: Standard deviation
